# Spatiotemporal Differentiation and Balance Pattern of Ecosystem Service Supply and Demand in the Yangtze River Economic Belt

**DOI:** 10.3390/ijerph19127223

**Published:** 2022-06-13

**Authors:** Chong Zhao, Pengnan Xiao, Peng Qian, Jie Xu, Lin Yang, Yixiao Wu

**Affiliations:** 1 School of Chemistry and Environmental Engineering, Wuhan Polytechnic University, Wuhan 430040, China; zhaochong426@whpu.edu.cn (C.Z.); 12683@whpu.edu.cn (L.Y.); yixiaowu@whu.edu.cn (Y.W.); 2 College of Urban and Environmental Sciences, Central China Normal University, Wuhan 430079, China; 3 School of Business Administration, Zhongnan University of Economics and Law, Wuhan 430073, China; yqian@stu.zuel.edu.cn; 4 Faculty of Resources and Environmental Science, Hubei University, Wuhan 430062, China; 201901110800086@stu.hubu.edu.cn

**Keywords:** ecosystem service supply, balance pattern, Yangtze River economic belt, ecosystem service demand, ecosystem services

## Abstract

Analyzing the supply and demand of ecosystem services and the regional balance pattern is an important basis for improving the ecological management level. Taking the Yangtze River economic belt as the study area, the spatiotemporal characteristics and balance pattern of ecosystem service supply and demand are quantitatively revealed based on equivalent factors, supply and demand balance modeling and quantile regression. The results show that: (1) the ecosystem services value in the research area experienced a change process of “increase–decrease–increase” from 2000 to 2020. The ecological service value of cultivated land and grassland presented a continuous decline, with decreases of 20.446 billion and 4.53 billion yuan, respectively, in the past 20 years, with reduction rates of −4.82% and −3.98%, respectively. (2) The demand for ecosystem services showed an unbalanced and phased growth trend. The total demand for ecosystem services showed heterogeneity and agglomeration effects in space. High demand and higher demand areas are mainly distributed in the regions with relatively developed population and economy, including Yangtze River Delta urban agglomeration, “Changsha–Zhuzhou–Xiangtan” urban agglomeration, Poyang Lake Plain, Jianghan Plain and Chengdu Plain. (3) The overall pattern of the supply–demand balance of ecosystem services has changed little; however, there have also been significant changes in certain areas in individual years.

## 1. Introduction

Ecosystem services (ES) refer to ecological products and services that mankind obtains through direct or indirect means [1], as well as environmental conditions and utilities that affect mankind’s continuation and prosperity [2]. The complex linkages between nature and mankind is manifested through the supply and demand of ecosystem services [3]. The supply of ecosystem services is considered to be the various benefits produced by the ecosystem for human beings [4], while the ecosystem services demand is considered to be the expenditure and utilization of the products and services supplied by the ecosystem [5], which together form the complex procedure of ecosystem products and services flowing from the natural environment to the socio-economic system [6,7,8]. Studying ecosystem services from the viewpoint of supply and demand can better promote the realization of ecological security and sustainable development of society and economy [9,10,11]. Identifying the supply areas of ecosystem services and assessing their supply potential will help to meet the growing social needs [12,13]. Clarifying the demand space and demand structure of ecosystem services will be helpful in exploring what impact ecosystem services have on economic development, including promotion and restriction [14,15]. Analyzing the differences between the supply and demand of ecosystem services can not only reflect the spatial allocation of environmental resources but also provide a new perspective for ecosystem service payment and ecological compensation [16]. However, against the backdrop of rapid urbanization, population migration and continuous expansion of impervious surface, the balance between the supply and demand of ecosystem services has been gradually broken, resulting in a series of environmental damage, including habitat loss, air pollution, reduction of ecological land and environmental damage. It will be helpful to promote the efficiency of ecosystem management by identifying, analyzing and calculating the supply–demand and the balance pattern of ecosystem services.

The current research mainly focuses on ecosystem service assessment [17,18,19], ecosystem service simulation prediction [20,21,22], driving mechanism [23,24], trade-off and coordination [25,26], human well-being [27,28] and ecosystem service supply and demand [10,14,15]. The research on the “supply–demand” balance of ecosystem services mainly concerns two aspects: First, the calculation and spatiotemporal representation of the ecosystem services in “supply–demand balance”. At present, the spatial methods of ecosystem service supply and demand are diverse and have their own advantages and disadvantages due to different research areas, objectives, objects and data [10,29,30,31]. Combing the existing literature, the spatial methods can be summarized into four categories: land use estimation [32,33], ecological process simulation [34,35], data spatial superposition [36,37] and expert experience discrimination [38]. In addition, as an integrated model integrating multiple algorithms into the same platform, InVEST (integrated valuation of ecosystem services and trade-offs) [39,40,41,42] and ARIES (artificial intelligence for ecosystem services) [43,44] have different development purposes and application scopes; however, they can select corresponding modules for spatial analysis for a variety of ecosystem service types, which have good development and application prospects [10]. Many scholars have studied the spatiotemporal evolution of the balance between the supply and demand of ecosystem services from various scales, such as national [45], city [36,46], county [47,48], Yangtze River economic belt [49], Yangtze River Delta urban agglomeration [29,50] and land senses [51]. Second is the application of the balance between the supply and demand of ecosystem services. The connection between the supply and demand of ecosystem services has been applied from the aspects of ecological network spatial optimization [30,31,52,53], land regulation spatial zoning [54,55,56,57], ecological security pattern [50,58,59,60,61] and land ecological restoration [62,63,64]. The existing research results have laid a certain foundation for exploring the pattern of balanced supply–demand relationship for ecosystem services; however, there are still some improvements. First, these results mostly depend on large scales, such as national, provincial and municipal levels, but pay less attention to the measurement of the spatiotemporal evolution of basin scales [65,66]. Second, they focus on the unilateral spatiotemporal succession of supply and demand; however, there are relatively few studies on the spatiotemporal evolution of both the supply and demand sides and the balance pattern of supply and demand [67,68]. Third, the research focuses on economically developed regions, including “Beijing–Tianjin–Hebei” urban Agglomeration and Yangtze River Delta urban agglomeration and pays less attention to the ecosystem services from the perspective of “supply–demand-balance” pattern in the research area [69,70,71].

With unique geographical environment and convenient land and water transportation conditions, the Yangtze River Economic Belt has gathered many modern industries and leading enterprises, such as steel, automobile, electronics and petrochemical, in China and has become an economic belt with the strongest economic development level and comprehensive competitiveness in China [72]. At the same time, it is also a priority area for biodiversity protection with more ecological security barriers and global significance. However, inadequate protection of the river basin has recently resulted in frequent ecological problems [54]. This study attempts to take the Yangtze River economic belt as the research area to select relevant data on land use type from 2000 to 2020 and adopt the methods of equivalent factor, supply–demand balance analysis and quantile regression to quantitatively analyze the supply–demand and balance pattern of ecosystem services in the research area to thus provide a decision-making basis to realize harmonious development between the economy and environment.

## 2. Materials and Methods

### 2.1. Research Area

**Figure 1:**
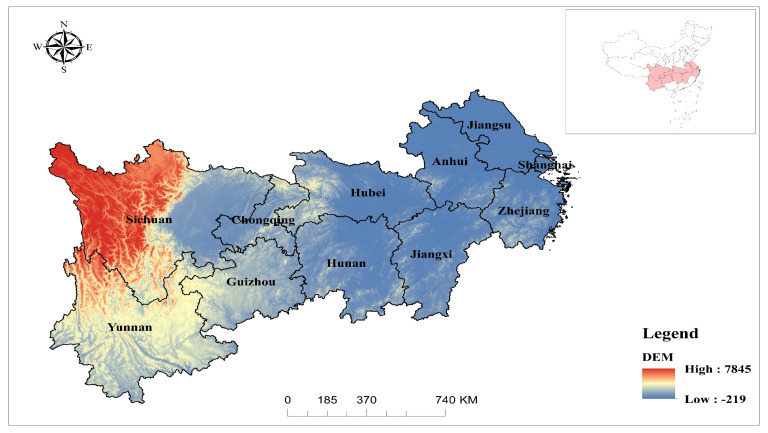
Location of the research area.

The research area consists of two municipalities and nine provinces, namely Chongqing, Jiangxi, Hubei, Hunan, Shanghai, Guangzhou, Yunnan, Sichuan, Anhui, Zhejiang and Jiangsu (see Figure 1). It covers an area of about 2.0523 million square kilometers, accounting for 21.4% of China’s total land area. The proportion of population and GDP (Gross Domestic Product) in the study area in China exceeds 40% [73]. With a subtropical monsoon climate, the region is characterized by sufficient precipitation and simultaneous rain and heat. The terrain of this area is undulating, complex and diverse, and the plateau and mountainous areas account for a large proportion. 

The Yangtze River economic belt, mainly composed of Sichuan Basin and the plains in the middle and lower reaches of the Yangtze River, is an important ecological security barrier area in China. It not only has many lakes, wetlands and relatively rich water resources but also has fragile ecological environment areas in many countries. The research area has formed some key areas and covered more than 50 prefecture level cities, including the Yangtze River Delta urban agglomeration, “Changsha–Zhuzhou–Xiangtan” urban agglomeration, Hefei metropolitan area, “Chengdu–Chongqing” urban agglomeration, Wuhan urban agglomeration, Central Yunnan Urban Agglomeration and central Guizhou urban agglomeration.

### 2.2. Data Source

The research data mainly include land use data, night light remote sensing data and economic data. Among the three types of data, the land use data comes from the resource and environment science data center of the Chinese Academy of Sciences (https://www.resdc.cn, accessed on 1 March 2022), with a resolution of 1 km, and the research periods are 2000, 2005, 2010, 2015 and 2020. By re-classifying the data of land use types each year, this study obtained six primary land use types (including cultivated land, forest land, grassland, water area, construction land and unused land).

**Figure 2:**
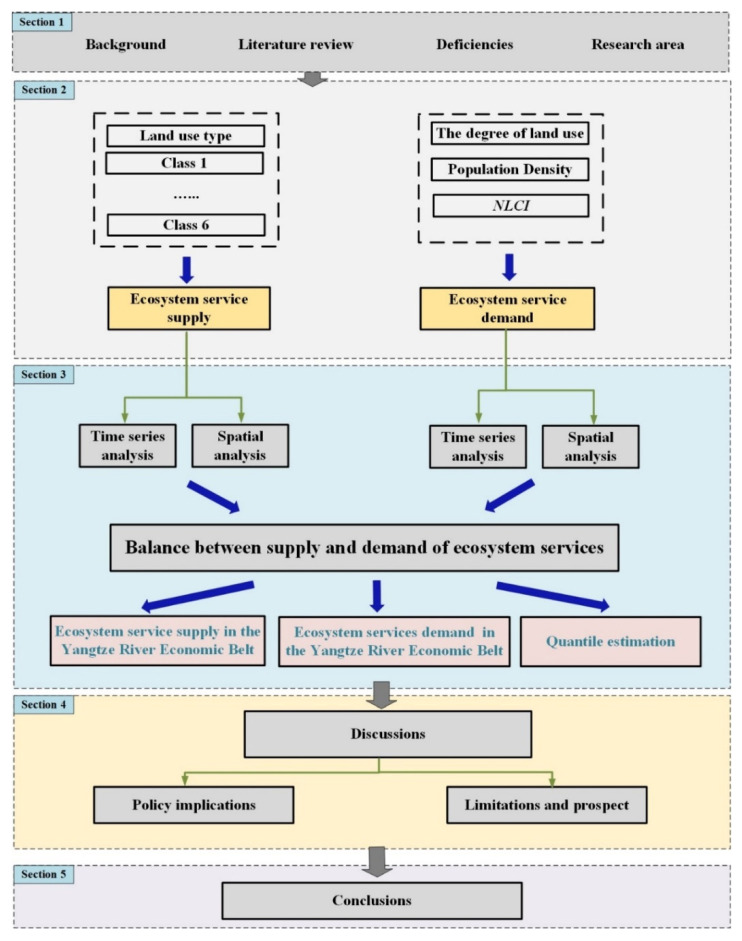
Technical flowchart of our research.

The population density data comes from the WorldPop data set with a 1 km resolution (https://www.worldpop.org/methods/populations, accessed on 1 March 2022). The nighttime light data comes from DMSP-OLS nighttime lights (https://www.ngdc.noaa.gov, accessed on 1 March 2022) and VIIRS nighttime lights (https://eogdata.mines.edu/product/vnl/, accessed on 1 March 2022). This study takes the county-level administrative units as the research unit for the analysis of the supply and demand and balance pattern of ecosystem services in the research area. Figure 2 is the technical route of the whole article.

### 2.3. Research Method

#### 2.3.1. Ecosystem Service Supply

Ecosystem service supply is the capability of ecosystem to supply specific ecological products and services in specific time and space [3]. Ecosystem services value (ESV) is an economic measure of the supply capacity, which can reflect the supply state of ecosystem services to an extent [74]. At present, scholars mainly use the functional value method [25,75] and the equivalent factor method [49,50] to compute the supply of ecosystem services. 

Among them, the equivalent factor method is relatively easy to use, the calculation steps are simplified, the required data are few, and the evaluation results are highly comparable. Hence, this method is frequently used in evaluating the supply of ecosystem services. Therefore, based on the relevant achievements of [76,77], this study obtains the ecological value coefficient of each land use type (see Table 1) to calculate the ecosystem service value of the study area. The formula is as follows:

(1) $$\;ESV=\sum \left(A_{k}\times VC_{k}\right)$$

where *ESV* is the ecosystem service value in the research area, unit: yuan/a; *A_k_* is the area of class *k* land use type in the research area, unit: hm^2^; and *VC_k_* is the ecosystem service value coefficient of class *k* land use type, unit: yuan/(hm^2^·a);

#### 2.3.2. Ecosystem Service Demand

Referring to the research of [3], this study applies the comprehensive index, composing of land use degree, population density and night light comprehensive index, to calculate the demand of ecosystem services [72]. The formula is as follows:

(2) $$\;X_{i}=D_{i}\times \log P_{i}\times \log NLCI_{i}$$

where *X* is the ecosystem service demand index; *D_i_* is the degree of land use (%); *P_i_* is the population density (person/km^2^); *NLCI_i_* represents the night light comprehensive index; and *i* is the serial number of the administrative region.

The degree of land use is expressed by the percentage of the built-up area in a certain area in the total land area [54]. Based on the research results of [78,79], the night light comprehensive index (*NLCI*) is calculated as follows:

(3) $$NLCI=P_{1}\times I+P_{2}\times S$$

where *I* is the average night light intensity of the study area, and *S* is the proportion of the light pixel area of the study area to the total pixel area of the study area. *P*_1_ and *P*_2_ represents the weights of *I* and *S*, which are 0.8 and 0.2, respectively.

The calculation formula of average night light intensity *I* is

(4) $$I=\frac{\sum_{j=1}^{63}\left(DN_{j}\times N_{j}\right)}{N_{t}\times 63}$$

where *DN_j_* is the pixel gray value of the *j* gray level. *N_j_* is the number of all pixels of the *j*-th gray level. *N_t_* is the total number of pixels with 63 ≤ *DN* ≤ 1.

The proportion of night light pixel area to the total pixel area *S* is calculated as follows:

(5) $$S=\frac{N_{t}}{N}$$

where *N_t_* is the number of all pixels with 63 ≤ *DN* ≤ 1; and *N* is the total number of pixels in the study area.

#### 2.3.3. Balance between the Supply and Demand of Ecosystem Services

On the basis of referring to the concept of the supply and demand of the ecological carrying capacity proposed by [80] and the construction method of comprehensive index of the supply and demand of ecosystem services proposed by [81], this study uses the supply and demand balance index of ecosystem services to reflect the supply and demand status of ecosystem services, including the surplus, deficit and balance. The formula is as follows:

(6) BI=ESV−X

where *BI* refers to the balance index of the supply and demand of ecosystem services, *ESV* refers to the supply of ecosystem services, and *X* refers to the demand of ecosystem services.

#### 2.3.4. Quantile Regression Analysis

Referring to the research of [47] and taking the supply–demand balance index of ecosystem service as the explained variable and the proportion of each land use as the explanatory variable, a quantile regression model is established to quantify the effect of each land use type with regard to the ecosystem service supply and demand balance index. Compared with ordinary least squares (OLS), quantile regression can measure the regression coefficient of the explained variable under different quantiles [82] and is less affected by extreme values. The formula is as follows:

(7) $$Q_{\theta }\left(Y|X\right)=X^{\prime }\beta \left(\theta \right)$$

where $Q_{\theta }\left(Y|X\right)$ is the value of the explained variable *Y* at the θ quantile when the explanatory variable *X* is clear. *X* = (urban land proportion, rural residential area proportion, other construction land proportion, paddy field proportion, dry land proportion, other forest land proportion, natural grassland proportion, river canal proportion, forest land proportion and lake proportion), and *X* is the vector of explanatory variables [47]. β(θ) is the regression coefficient vector at locus θ, which meets the following requirements [82]:

(8) $$\beta \left(\theta \right)=min\left\{\underset{i:Y\geq X^{\prime }}{\sum }\theta \left|yY-X^{\prime }\beta \right|+\underset{i:Y<X^{\prime }\beta \left(\theta \right)}{\sum }\left(1-\theta \right)\left|Y-X^{\prime }\beta \left(\theta \right)\right|\right\}$$

With the value of θ from 0 to 1, this method can describe the conditional distribution trajectories of all *Y* on *X*. This study gives the parameter estimation results at 0.1, 0.3, 0.5, 0.7 and 0.9 quantile to analyze the role of various land use types on the supply–demand balance index of different ecosystem services.

## 3. Results

### 3.1. Changes of Land Use Types

**Figure 3:**
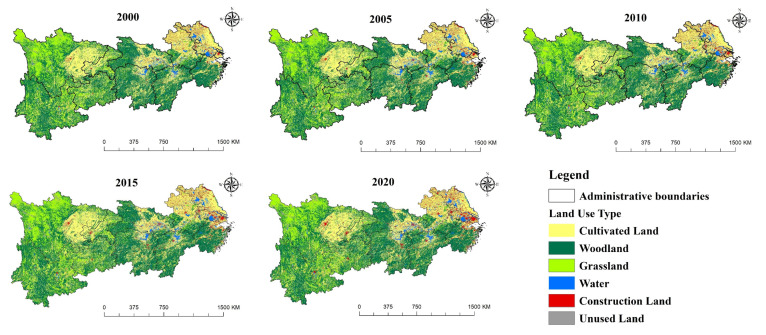
Land use types of the Yangtze River Economic Belt (2000–2020).

As can be seen from Figure 3 and Table 2, according to the land use data, the cultivated land area of this region decreased the most from 2000 to 2020, with a decrease of 30,768 km^2^. In addition, the growth rate of construction land area ranked first among various land use type, with an increase of 35,687 km^2^. The water area increased by 5732 km^2^. Forest land increased by 2156 km^2^, grassland decreased by 13,599 km^2^, and the area of unused land changed little. In terms of growth rate, from 2015 to 2020, the growth rate of construction land area is the largest, as high as 21.86%. The cultivated land, forest land, grassland and water also changed with the growth rates of −1.82%, 0.36%, −3.37% and 4.26%, respectively. In terms of phased changes, the cultivated land and grassland area has been decreasing with different scales. The water and construction land continue to grow steadily, and the unused land shows a wave type of “decrease–increase–decrease”.

### 3.2. Ecosystem Services Supply

**Figure 4:**
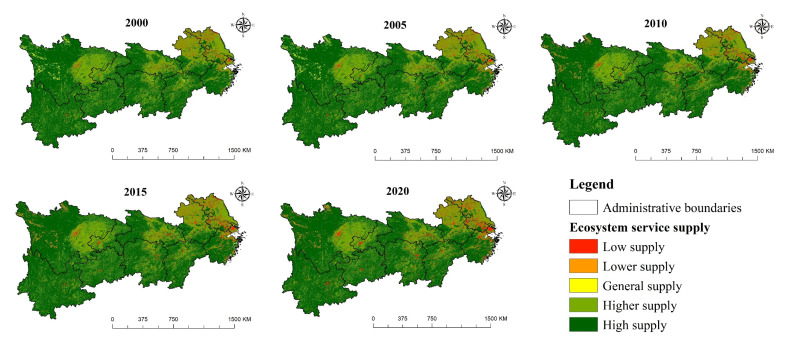
The ecosystem service supply in the Yangtze River Economic Belt (2000–2020).

In ArcGIS 10.7 software (ESRI, Redlands, CA, USA), the ecosystem services supply of the Yangtze River Economic Belt is divided into five levels according to the natural breakpoint method, including high supply, higher supply, general supply, lower supply and low supply. Figure 4 and Table 3 show that the total value of ecosystem services in research area experienced a dynamic process of “increase–decrease–increase” from 2000 to 2020. It increased from 2890.479 billion yuan in 2000 to 2894.590 billion yuan in 2005, an increase of 4.111 billion yuan, with a growth rate of 0.14%, then decreased to 2893.758 billion yuan in 2010, a decrease of 832 million yuan, with a reduction rate of −0.03%. Subsequently, it continued to decrease to 2887.273 billion yuan in 2015, a decrease of 6.484 billion yuan, with a reduction rate of −0.22% and finally increased by 3.198 billion yuan, reaching 2890.472 billion yuan in 2020, with a growth rate of 0.11%. The ecosystem services value of cultivated land and grassland showed a trend of continuous decline, with decreases of 20.446 billion and 4.53 billion yuan, respectively, in the last 20 years, with reduction rates of −4.82% and −3.98% respectively. After experiencing the change process of “increase–decrease–increase”, the ecosystem service value of unused land increased by 3 million yuan. The ecosystem service value of the water area has continued to grow, with an increase of 25.363 billion yuan in recent 20 years, with a growth rate of 9.90%.

Table 4 shows that in the dynamic change of single ecosystem service value from 2000 to 2005, except for the increase of ecosystem service value of water conservation, waste treatment, raw materials and entertainment, the remaining single ecosystem service value reflects the reduction in varying degrees. Among them, gas regulation, climate regulation, soil formation and protection, biodiversity protection and food production decreased by 1.8 billion, 2.994 billion, 6.056 billion, 1.479 billion and 3.275 billion yuan, respectively. Over time, the value of gas regulation, climate regulation, soil formation and protection and food production decreased continuously from 2000 to 2020. The value of water conservation and entertainment continues to increase due to the overall growth of wetland and water area. Waste treatment and raw materials go through an “increase–decrease–increase” process. From the composition of ecosystem service value, the proportion of individual ecosystem service value is as follows: soil formation and protection > water conservation > biodiversity protection > gas regulation > waste treatment > climate regulation > raw materials > entertainment > food production. 

Among them, the soil formation and protection and water conservation account for a large proportion, accounting for more than 15% of the total value of each year. Including biodiversity protection, gas regulation, waste treatment and climate regulation, the sum of the four is basically maintained at more than 40% of the total value of each year. The food production value is the lowest, accounting for no more than 3% of the total value of ecosystem services per year. The sum of the six functional values, including waste treatment, biodiversity protection, soil formation and protection, water conservation, climate regulation and gas regulation, accounts for more than 80% of the total value of ecosystem services in each year.

### 3.3. Ecosystem Services Demand

**Figure 5:**
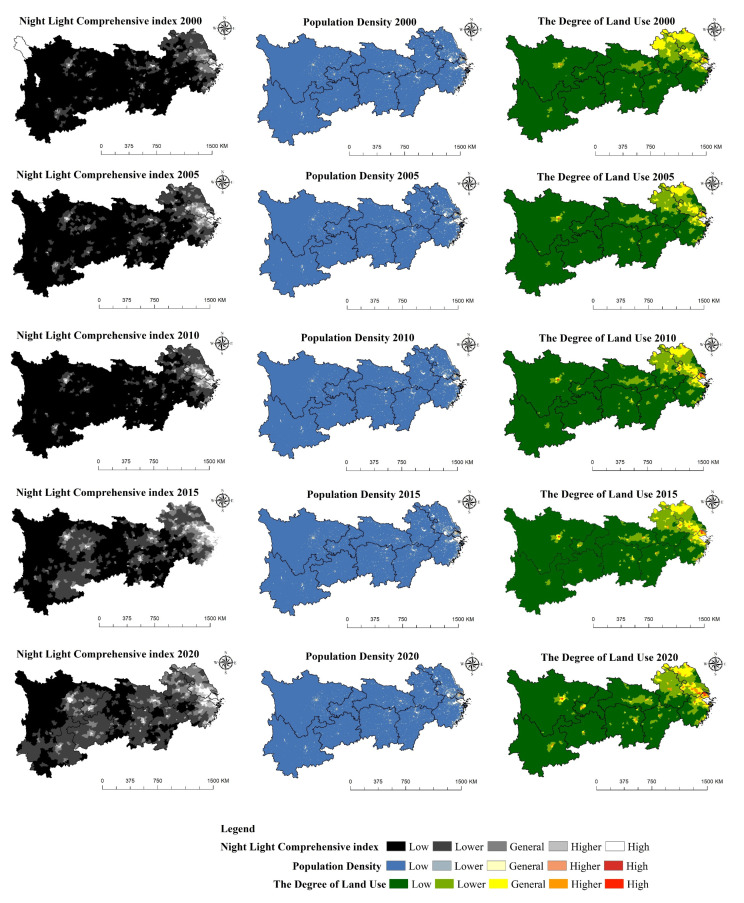
Sub-item demand for ecosystem services in the Yangtze River Economic Belt (2000–2020).

In ArcGIS 10.7, this paper divides the night light comprehensive index, population density and land use degree into five levels based on the natural breakpoint method. In Figure 5, the comprehensive index of night light, population density and land use degree show relatively consistent change characteristics—that is, unbalanced development and a distinct urban agglomeration effect. The unbalanced development is manifested as “higher in the East and lower in the west, higher in the coast and lower in the interior”. The overall development level of the eastern region is higher than that of the central and western regions, and the regional urbanization level of the coastal zone is much higher than that of the inland region. In particular, the spatial distribution characteristics of night light comprehensive index are very clear. In 2000, there was still a data gap in the northwest of Sichuan Province, indicating that human economic activities were not distinct yet. By 2020, the areas with data gaps had large traces of human activities. This also shows the superiority of using the night light index instead of the GDP per area to express the economic density, which can express the intensity of economic activities more clearly. The urban agglomeration effect is reflected in that, throughout the study period, the Yangtze River Delta urban agglomeration, northern Jiangsu, Chengdu Plain, Hefei urban circle, Central Yunnan Urban Agglomeration, Poyang Lake Plain, “Changsha–Zhuzhou–Xiangtan” urban agglomeration and Jianghan Plain show the characteristics of strong economic activity, population concentration and high land use degree, and the general area, low value area and lower value area are surrounded around the high value area and higher value area.

**Figure 6:**
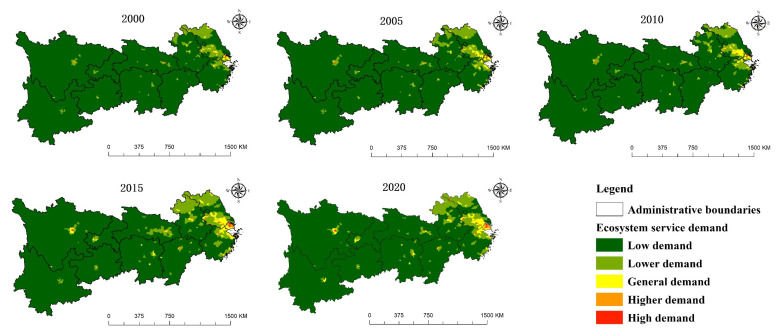
Spatial distribution of the ecosystem services demand (2000–2020).

According to the natural breakpoint method, the ecosystem services demand is divided into five levels in ArcGIS 10.7, namely, high demand, higher demand, general demand, low demand and lower demand. Figure 6 show that the overall demand for ecosystem services from 2000 to 2020 reflects the characteristics of unbalanced and phased growth. From 2000 to 2005, the demand of Yangtze River Delta urban agglomeration, Sichuan Basin, northern Jiangsu and Wuhan urban circle increased rapidly. From 2005 to 2010, the growth of the overall demand in the research area was not obvious; however, the demand of some urban agglomerations in the Yangtze River Delta is still expanding. From 2010 to 2015, the demand growth of Chongqing, Chengdu, Wuhan, Kunming, “Changsha–Zhuzhou–Xiangtan” urban agglomeration, Nanchang and southern Zhejiang is relatively clear. The total demand for ecosystem services shows the characteristics of heterogeneity and agglomeration in space, which is mainly reflected in that the high demand and higher demand areas are mainly areas with relatively developed population and economy, such as Chengdu Plain, Yangtze River Delta urban agglomeration, Poyang Lake Plain, “Changsha–Zhuzhou–Xiangtan” urban agglomeration and Jianghan Plain. 

The periphery of high demand and high demand is surrounded by a general demand area, low demand area and lower demand area. Considering that China is still in the process of urbanization and population urbanization, economic urbanization and geospatial urbanization are particularly obvious in economically developed provincial capital cities and urban agglomerations, and the demand for ecosystem services in these areas is increasing. The economy of the periphery of the economic center and areas with relatively poor geographical environment is comparatively backward, resulting in lower population and low demand for ecosystem services.

### 3.4. Balace Pattern between the Supply and Demand of Ecosystem Services

**Figure 7:**
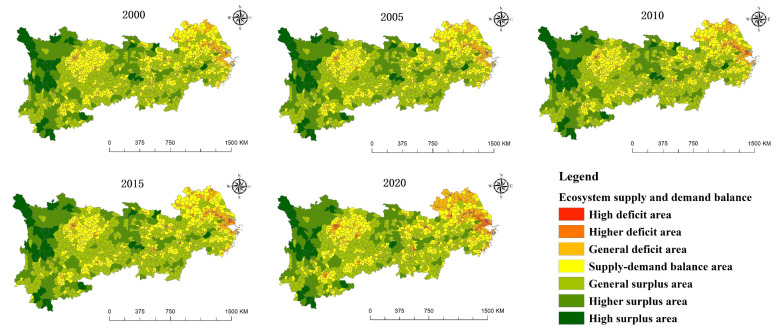
Ecosystem services supply and demand balance.

The supply–demand balance index of ecosystem services can roughly reflect the balance between the supply and demand of ecosystem services in the research area [50]. The natural breakpoint method applied in ArcGIS divides the region into seven types of areas: high surplus area, higher surplus area, general surplus area, high deficit area, higher deficit area, general deficit area and supply–demand balance area [54] (as shown in Figure 7). 

With regard to time, the distribution of the supply–demand balance changed slightly from 2000 to 2020; however, there were clear fluctuations in some areas in individual years. The dimension of the high deficit area was not shrinking but expanding. In the Yangtze River Delta urban agglomeration, Chengdu, Kunming, “Changsha–Zhuzhou–Xiangtan” urban agglomeration and Wuhan, there are many general deficit areas transformed into high deficit areas and higher deficit areas, reflecting the imbalanced pressure of ecosystem services in these areas. In the future development, such areas should increase ecological land reasonably and control the unlimited expansion of construction land. In terms of spatial change, high surplus areas and higher surplus areas are distributed in western Sichuan, southern Sichuan, southwestern Sichuan, southern Yunnan, southeast Yunnan, western Hunan, southwestern Hubei and northwestern Hubei. These areas are the key areas of ecological protection, and with smaller populations, the supply of ecosystem services is in these areas are far greater than the demand, which results in a higher surplus. Generally, surplus areas are mostly located in the periphery of higher surplus areas and mostly in the transition zone of terrain, and the supply of ecosystem services is slightly greater than the demand in these areas. 

The supply–demand balance areas are mainly distributed in the middle and lower reaches of the Yangtze River Plain, Sichuan Basin, Dongting Lake Plain and Poyang Lake Plain. High deficit areas and higher deficit areas are mainly distributed in the Yangtze River Delta, as well as Chongqing, Chengdu, Changsha, Wuhan, Kunming, Guizhou and Nanchang. These areas have a concentrated population, prosperous economy and high level of urbanization. The demand for ecosystem services in these areas is far greater than the supply. By 2020, the general deficit areas will be mainly distributed around the Yangtze River Delta Urban Agglomerations, northern Anhui and northern Jiangsu.

### 3.5. The Relationship between the Spatial Pattern of the Supply and Demand of Ecosystem Services

By using Stata 15.0 software (Statacorp, College Station, TX, USA), quantile regression analysis of impact ratio of various land use types on the supply and demand balance index of ecosystem services in the Yangtze River Economic Belt was conducted (Table 5). OLS estimation results show that, except for other woodlands, the estimation coefficients of various living and production land are significantly negative at the level of 1%, indicating that living and production land have a significant negative impact on sustaining the supply–demand balance—in other words, the expansion of living and production land is not conducive to a higher level supply–demand balance. 

**Figure 8:**
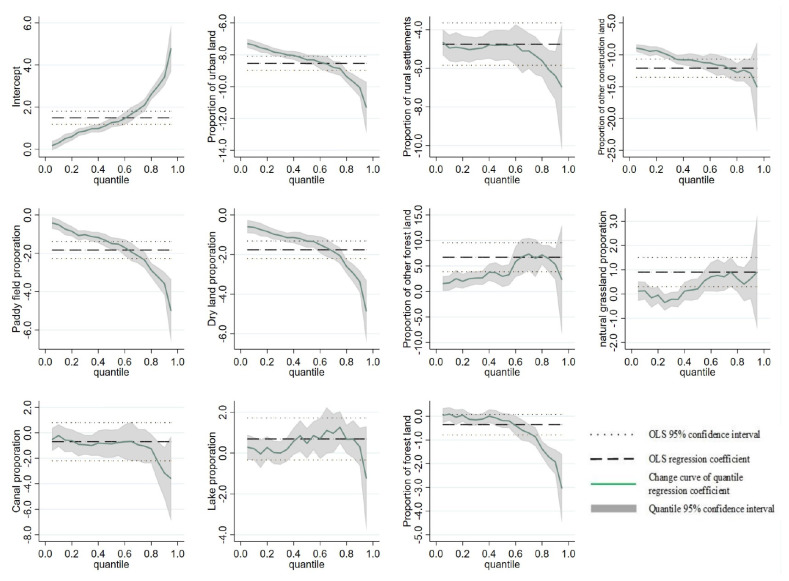
Parameter changes of various land use proportions under different quantiles in the quantile regression model.

The higher the proportion of living and production land, the lower the supply–demand balance. The coefficient of other forest land and natural grassland to the supply–demand balance degree of ecosystem is significantly positive, indicating that the higher the proportion of other forest land and natural grassland, the higher the supply–demand balance degree of the ecosystem. The quantile regression results (Table 5 and Figure 8) show that, among living and production land, other construction land, urban land, rural residential areas, paddy fields and dry lands have different negative impacts on the balance of ecosystem services. The higher the degree of equilibrium, the greater the negative effect. The relationship between other woodlands and the supply–demand balance showed a roughly inverted U-shape of first increasing and then decreasing. On the whole, no matter the mean effect or at each quantile, the living and production land (except other forest land) had a significant negative impact on the supply–demand balance. There was a positive relationship between other forest lands and natural grassland on the supply–demand balance of ecosystem services.

## 4. Discussion

### 4.1. Deficiency and Prospect

Based on the research of [2,76] and referring to the research of [74,76,77], the research results basically accord with the distribution pattern of ecosystem service value calculated by [49,50], which indicates that the calculation results of this study are relatively reasonable. However, this study did not further explore the mechanism of habitat quality, topographic potential index, spatial accessibility and other factors on ecosystem service supply and did not distinguish and quantify the actual supply and potential supply of ecosystem service. The distinction between the potential supply and actual supply reflects the sustainable development state of ecosystem, furthermore, the distinction between the potential demand and actual demand reflects the extent to which social needs are met [14]. In future in-depth research, we can further consider the role of other factors on ecosystem service supply and explore the actual supply and potential supply to thus make the research more in-depth. This study draws on and improves the estimation system of ecosystem service demand proposed by [47,76,83] and comprehensively measures the ecosystem service demand by land use and development degree, population density and night light comprehensive index. After replacing the original economic density with the night light comprehensive index, the calculation process is simpler, and the reliability of the result is higher. This method does not take the preferences and expectations of the demand subject into consideration and cannot analyze the actual demand and potential demand. 

In future research, multi-source data and multidisciplinary methods should be used to identify multiple needs to thus make the results more credible. At present, many scholars mostly use an ecosystem service supply and demand matrix [84] and the quadrant method [54] to measure the balance pattern. After referring to the results of [81], this study uses the difference between supply and demand to characterize the balance between two sides, however, does not further consider the impact of spatial flow of ecosystem services in the study area. Various models can be applied to study the services’ flow on the “supply side” and “demand side” in future research. Ecosystem services can be produced on different temporal and spatial scales, with the characteristics of “time dimension” and “spatial scale” [85]. Different temporal and spatial scales will lead to different balance and matching patterns [86,87], trade-offs and synergies [88,89] and “driving–response–impact” mechanisms [90]. Therefore, the research on the supply and demand of ecosystem services needs to consider different time dimensions and spatial scales. At present, a large amount of ecosystem services monitoring work has been conducted worldwide and has played a strategic guiding role in the research on the supply and demand of ecosystem services; however, these works have not reached ideal results in resource protection [91]. This is mainly because the ecosystem corresponding to the global scale is complex, and the response cycle is long when it is disturbed by the external environment [92]. 

Therefore, most current research on ecosystem services mainly focuses on the specific quantitative calculation of supply and demand and the analysis of spatial matching characteristics. The supply side should not only satisfy the current demand for ecosystem services but also meet the needs of future generations in the long run [46]. In the future, when studying the supply and demand of global and national large-scale ecosystem services, we should take a longer view from a sustainability perspective. At the regional level, the current research on ecosystem services focuses on the coordination of supply and demand [93]. The spatial utilization layout of resource elements in the region will impact the production and transportation of ecosystem services, end up with significant changes in the trade-off/synergy between various services [94]. However, at the regional level, the ecosystem change cycle is long, and the changes of ecosystem service supply and demand should be considered in the long run. At the small-scale landscape level, the research on ecosystem services from the supply and demand perspective needs to focus on applications [95]. 

Considering the strong availability of landscape level data, large-scale and high-resolution data can be selected in studies [96]. Due to the low resistance of the ecosystem at the landscape level to external interference and the effects of some changes in natural factors, land use and socio-economic factors on the supply and demand of ecosystem services, it is suitable to explore the influence mechanism of ecosystem service supply and demand at this scale [97,98]. Considering that the ecosystem change cycle at the landscape level is short, the frequency is high, and it is prone to mutation, this can correspond to a shorter time dimension.

### 4.2. Policy Enlightenment

Evaluating the supply and demand of ecosystem services is not the main purpose. The research value lies in incorporating the evaluation results into the management decision-making process and maintaining the stability of the ecosystem while improving human well-being as much as possible [14,99].

In this study, the surplus area included three types: a high surplus area, higher surplus area and general surplus area, and most of these areas are within the scope of ecological protection. The ecological environment is relatively good, the population distribution is relatively scattered, and there is less interference caused by human activities in these areas. Therefore, the government should strictly implement the ecological and environmental protection policy, emphasize the comprehensive protection of nature resources, enhance the capacity of soil and water conservation, transform the mode of economic development and realize the sustainable development of human and nature.

There are mostly concentrated and contiguous cultivated land in the supply–demand balance area; however, there is also the possibility of imbalance in these areas. Therefore, special attention should be paid to the coordinated development between human and nature. Attention should also be paid to the optimization of the spatial layout of residential areas and the improvement of the scale and quality of agricultural land. Under the premise of controlling the expansion of rural construction land, the stock of rural construction land should be revitalized and the structure of rural construction land should be improved. Through the construction of high standard basic farmland with guaranteed income in drought and flood and the improvement of farmland water conservancy infrastructure, a national strategic reserve grain production area will be formed.

The deficit areas, including high deficit areas, higher deficit areas and general deficit areas, are concentrated and have a high level of economic development, most of which are located in provincial administrative centers and urban agglomerations, and it is difficult for their ecosystem service supply to meet the increasingly diverse needs of urbanization and carbon neutrality. In future development, these regions must make efficient use of the land, promote comprehensive land improvement, control the expansion intensity of construction land, promote the intensive use of urban residential land, build an urban green space system and strengthen the connection between urban green space, wetland park and country parks and various ecological spaces.

## 5. Conclusions

Referring to the land use data, population density, night light and other socio-economic data in 2000, 2005, 2010, 2015 and 2020, this study comprehensively applied the equivalent factor method, supply and demand balance modeling and quantile regression to explore the supply–demand-balance pattern of ecosystem services in the research area. The main conclusions can be drawn as follows:

(1) From 2000 to 2020, the cultivated land area decreased the most, with a decrease of 30,768 km^2^. The area growth of construction land ranked first among various land use types, with an increase of 35,687 km^2^. The water increased greatly, with an increase of 5732 km^2^. Forest land increased by 2156 km^2^, grassland decreased by 13,599 km^2^, and the area of unused land changed little. In the time period, the total value of ecosystem services experienced a change process of “increase–decrease–increase”, the ecological service value of cultivated land and grassland presented a trend of continuous decrease, the unused land experienced a change process of “increase–decrease–increase”, and the ecological service value of water areas continued to grow.

(2) The ecosystem services demand showed the characteristics of unbalanced and phased growth. The total demand for ecosystem services showed heterogeneity and agglomeration effects in space. High demand and higher demand areas were mainly distributed in the Yangtze River Delta, the middle and lower reaches of the Yangtze River Plain, “Changsha–Zhuzhou–Xiangtan” urban agglomeration and Chengdu Plain with a relatively developed population and economy.

(3) From 2000 to 2020, the overall pattern of the supply–demand balance of ecosystem services changed slightly; however, there were also clear fluctuations in certain areas in individual years. The scope of the high deficit area was not shrinking but expanding.

(4) It can be seen that the influencing factors of the ecosystem supply–demand balance were significantly different at different quantiles. Regardless of the mean effect or at each quantile, living and production land (except other forest land) had a significant negative impact on the balance between the supply and demand of ecosystem services. Other forest land and natural grassland had positive impacts on the balance of the supply and demand of ecosystem services.

## Figures and Tables

**Table 1:** Ecological value coefficient of land use types.

Primary Ecological Service	Secondary Ecological Services	Cultivated Land	Woodland	Grassland	Water	Unused Land	Construction Land
Regulation service	Gas regulation	480.85	3365.95	769.36	0	0	0
	Climate regulation	855.91	2596.59	856.53	442.38	0	0
	Water conservation	577.02	3077.44	769.36	19,618.68	28.85	0
	Waste disposal	1577.19	1259.83	1259.83	17,502.94	9.62	0
Support services	Soil formation and protection	1404.08	3750.63	1875.32	9.62	19.23	0
	Biodiversity conservation	682.81	3135.14	1048.25	2394.63	326.98	0
Supply service	Food production	961.7	96.17	288.51	96.17	9.62	0
	Raw material	96.17	2500.42	48.08	9.62	0	0
Cultural Services	Entertainment	9.62	1230.98	38.47	4173.78	9.62	0

**Table 2:** Changes of land use types in the Yangtze River Economic Belt (2000–2020).

Land Use Type		Cultivated Land	Woodland	Grassland	Water	Construction Land	Unused Land
Area (km^2^)	2000 Year	638,571	938,340	341,406	57,877	47,197	21,814
	2005 Year	632,019	939,466	339,984	59,481	52,900	21,585
	2010 Year	627,056	939,880	339,431	59,925	57,453	21,787
	2015 Year	619,095	937,085	339,253	61,011	68,015	21,846
	2020 Year	607,803	940,496	327,807	63,609	82,884	21,766
Area change	2000–2005 (km^2^)	−6552	1126	−1422	1604	5703	−229
	2000–2005 (%)	−1.03%	0.12%	−0.42%	2.77%	12.08%	−1.05%
	2005–2010 (km^2^)	−4963	414	−553	444	4553	202
	2005–2010 (%)	−0.79%	0.04%	−0.16%	0.75%	8.61%	1.33%
	2010–2015 (km^2^)	−7961	−2795	−178	1086	10,562	59
	2010–2015 (%)	−1.27%	−0.30%	−0.05%	1.81%	18.38%	0.15%
	2015–2020 (km^2^)	−11,292	3411	−11,446	2598	14,869	−80
	2015–2020 (%)	−1.82%	0.36%	−3.37%	4.26%	21.86%	−0.40%
	2000–2020 (km^2^)	−30,768	2156	−13,599	5732	35,687	3
	2000–2020 (%)	−4.82%	0.23%	−3.98%	9.90%	75.61%	0.01%

**Table 3:** Changes in the ecosystem service supply in the Yangtze River Economic Belt (2000–2020).

Land Use Type		Cultivated Land	Woodland	Grassland	Water	Construction Land	Unused Land	Total
ESV (100 million yuan)	2000 Year	4243.53	19,717.48	2374.04	2560.93	0	8.81	28,904.79
	2005 Year	4199.99	19,741.14	2364.15	2631.90	0	8.72	28,945.90
	2010 Year	4167.01	19,749.84	2360.30	2651.55	0	8.88	28,937.58
	2015 Year	4114.10	19,691.11	2359.07	2699.60	0	8.85	28,872.73
	2020 Year	4039.06	19,762.78	2279.47	2814.56	0	8.84	28,904.72
ESV Change	2000–2005	−43.54	23.66	−9.89	70.97	0	−0.09	41.11
	2000–2005 (%)	−1.03%	0.12%	−0.42%	2.77%	0	−1.05%	0.14%
	2005–2010	−32.98	8.70	−3.85	19.65	0	0.16	−8.32
	2005–2010 (%)	−0.79%	0.04%	−0.16%	0.75%	0	1.81%	−0.03%
	2010–2015	−52.90	−58.73	−1.24	48.05	0	−0.02	−64.84
	2010–2015 (%)	−1.27%	−0.30%	−0.05%	1.81%	0	−0.26%	−0.22%
	2015–2020	−75.04	71.68	−79.59	114.96	0	−0.02	31.98
	2015–2020 (%)	−1.82%	0.36%	−3.37%	4.26%	0	−0.18%	0.11%
	2000–2020	−204.46	45.30	−94.56	253.63	0	0.03	−0.07
	2000–2020 (%)	−4.82%	0.23%	−3.98%	9.90%	0	0.30%	0.00%

**Table 4:** Changes in the supply of individual services in the ecosystem (2000–2020).

Ecosystem Service Function		Gas Regulation	Climate Regulation	Water Conservation	Soil Formation and Protection	Waste Disposal	Biodiversity Conservation	Food Production	Raw Material	Entertainment	Total
ESV (100 million yuan)	2000	3728.13	3301.07	4654.92	5057.19	3632.64	3881.46	808.63	2424.63	1416.13	28,904.79
	2005	3727.67	3297.88	4684.97	5049.56	3650.00	3882.79	802.18	2426.76	1424.09	28,945.90
	2010	3726.26	3294.44	4691.68	5043.12	3649.78	3881.25	797.34	2427.30	1426.40	28,937.58
	2015	3712.89	3280.69	4699.65	5021.14	3652.49	3869.47	789.46	2419.55	1427.40	28,872.73
	2020	3710.13	3271.23	4745.79	4996.63	3670.03	3866.67	775.88	2426.47	1441.89	28,904.72
ESV Change	2000–2005	−0.45	−3.19	30.05	−7.63	17.37	1.33	−6.45	2.13	7.96	41.11
	2000–2005 (%)	−0.01%	−0.10%	0.65%	−0.15%	0.48%	0.03%	−0.80%	0.09%	0.56%	0.14%
	2005–2010	−1.41	−3.44	6.71	−6.44	−0.22	−1.53	−4.84	0.54	2.30	−8.32
	2005–2010 (%)	−0.04%	−0.10%	0.14%	−0.13%	−0.01%	−0.04%	−0.60%	0.02%	0.16%	−0.03%
	2010–2015	−13.38	−13.75	7.97	−21.99	2.70	−11.79	−7.87	−7.75	1.01	−64.84
	2010–2015 (%)	−0.36%	−0.42%	0.17%	−0.44%	0.07%	−0.30%	−0.99%	−0.32%	0.07%	−0.22%
	2015–2020	−2.76	−9.46	46.14	−24.50	17.54	−2.80	−13.58	6.92	14.49	31.98
	2015–2020 (%)	−0.07%	−0.29%	0.98%	−0.49%	0.48%	−0.07%	−1.72%	0.29%	1.02%	0.11%
	2000–2020	−18.00	−29.84	90.88	−60.56	37.39	−14.79	−32.75	1.84	25.76	−0.07
	2000–2020 (%)	0.00	−0.01	0.02	−0.01	0.01	0.00	−0.04	0.00	0.02	0.00

**Table 5:** Quantile estimation results of various land use proportions.

Explanatory Variable		OLS	Quantile				
			0.1	0.3	0.5	0.7	0.9
Intercept		1.494 ***	0.298 ***	0.866 ***	1.264 ***	1.879 ***	3.420 ***
		−0.159	−0.099	−0.13	−0.148	−0.196	−0.321
Living land	Proportion of urban land	−8.535 ***	−7.392 ***	−7.891 ***	−8.316 ***	−8.790 ***	−10.060 ***
		−0.224	−0.139	−0.184	−0.208	−0.277	−0.452
	Proportion of rural settlements	−4.749 ***	−4.947 ***	−4.989 ***	−4.793 ***	−5.098 ***	−6.382 ***
		−0.559	−0.347	−0.457	−0.518	−0.69	−1.127
Production land	Proportion of other construction land	−12.105 ***	−9.135 ***	−10.140 ***	−10.974 ***	−11.703 ***	−12.873 ***
		−0.727	−0.451	−0.594	−0.674	−0.897	−1.465
	Paddy field proportion	−1.827 ***	−0.507 ***	−1.027 ***	−1.489 ***	−2.133 ***	−3.582 ***
		−0.227	−0.141	−0.186	−0.21	−0.28	−0.457
	Dry land proportion	−1.757 ***	−0.622 ***	−1.038 ***	−1.322 ***	−1.843 ***	−3.369 ***
		−0.226	−0.14	−0.185	−0.21	−0.279	−0.456
	Proportion of other forest land	6.704 ***	1.687 *	2.623 **	2.966 **	7.334 ***	5.326 *
		−1.444	−0.895	−1.18	−1.338	−1.781	−2.909
Ecological land	Natural grassland proportion	0.902 ***	0.133	−0.214	0.209	0.709 *	0.626
		−0.306	−0.19	−0.25	−0.284	−0.377	−0.616
	Canal proportion	−0.694	−0.207	−0.93	−0.865	−0.934	−3.152 **
		−0.767	−0.475	−0.627	−0.71	−0.945	−1.544
	Lake proportion	0.689	0.219	0	0.47	0.966	0.308
		−0.524	−0.325	−0.428	−0.486	−0.646	−1.056
	Proportion of forest land	−0.355	0.091	−0.15	−0.185	−0.708 ***	−1.926 ***
		−0.221	−0.137	−0.181	−0.205	−0.273	−0.445

*, **, and *** denote statistical significance at 10%, 5%, and 1%, respectively.

## Data Availability

Not applicable.
